# Preoperative assessment of lymph node metastasis in Colon Cancer patients using machine learning: a pilot study

**DOI:** 10.1186/s40644-020-00308-z

**Published:** 2020-04-25

**Authors:** Aydin Eresen, Yu Li, Jia Yang, Junjie Shangguan, Yury Velichko, Vahid Yaghmai, Al B. Benson, Zhuoli Zhang

**Affiliations:** 1 Department of Radiology, Feinberg School of Medicine, Northwestern University, 737 N. Michigan Ave, Suite 1600, Chicago, IL 60611 USA; 2 Department of Gastrointestinal Surgery, The Affiliated Hospital of Qingdao University, Qingdao, Shandong China; 3 Department of Radiological Sciences, School of Medicine, University of California, Irvine, CA USA; 4 Robert H. Lurie Comprehensive Cancer Center of Northwestern University, 675 N. St. Clair, 21st Floor, Chicago, IL 60611 USA; 5 Division of Hematology and Oncology, Feinberg School of Medicine, Northwestern University, Chicago, IL USA

**Keywords:** Colon cancer, Computed tomography, Machine learning, Metastatic lymph node, Texture analysis

## Abstract

**Background:**

Preoperative detection of lymph node (LN) metastasis is critical for planning treatments in colon cancer (CC). The clinical diagnostic criteria based on the size of the LNs are not sensitive to determine metastasis using CT images. In this retrospective study, we investigated the potential value of CT texture features to diagnose LN metastasis using preoperative CT data and patient characteristics by developing quantitative prediction models.

**Methods:**

A total of 390 CC patients, undergone surgical resection, were enrolled in this monocentric study. 390 histologically validated LNs were collected from patients and randomly separated into training (312 patients, 155 metastatic and 157 normal LNs) and test cohorts (78 patients, 39 metastatic and 39 normal LNs). Six patient characteristics and 146 quantitative CT imaging features were analyzed and key variables were determined using either exhaustive search or least absolute shrinkage algorithm. Two kernel-based support vector machine classifiers (patient-characteristic model and radiomic-derived model), generated with 10-fold cross-validation, were compared with the clinical model that utilizes long-axis diameter for diagnosis of metastatic LN. The performance of the models was evaluated on the test cohort by computing accuracy, sensitivity, specificity, and area under the receiver operating curve (AUC).

**Results:**

The clinical model had an overall diagnostic accuracy of 64.87%; specifically, accuracy of 65.38% and 62.82%, sensitivity of 83.87% and 84.62%, and specificity of 47.13% and 41.03% for training and test cohorts, respectively. The patient-demographic model obtained accuracy of 67.31% and 73.08%, the sensitivity of 62.58% and 69.23%, and specificity of 71.97% and 76.23% for training and test cohorts, respectively. Besides, the radiomic-derived model resulted in an accuracy of 81.09% and 79.49%, sensitivity of 83.87% and 74.36%, and specificity of 78.34% and 84.62% for training and test cohorts, respectively. Furthermore, the diagnostic performance of the radiomic-derived model was significantly higher than clinical and patient-demographic models (*p* < 0.02) according to the DeLong method.

**Conclusions:**

The texture of the LNs provided characteristic information about the histological status of the LNs. The radiomic-derived model leveraging LN texture provides better preoperative diagnostic accuracy for the detection of metastatic LNs compared to the clinically accepted diagnostic criteria and patient-demographic model.

## Background

Colon cancer (CC) is a leading cause of cancer morbidity and mortality in the world with more than one million new cases in 2018 [1]. Despite advancements in treatment options of this disease, standard curative treatment is still complete resection of the primary tumor with dissection of regional lymph nodes (LNs) [2]. The presence of LN metastases plays a crucial role in the management and treatment strategy in CC [3, 4]. In clinical practice, preoperative identification of the histological status of LNs provides the basis for accurate planning of surgery, which not only ensures the quality and quantity of LN dissection but also avoids the omission of suspected LNs. Also, the presence of metastatic LNs determines the potential benefit of neoadjuvant chemotherapy in select CC patients. The recent Foxtrot trial investigated the potential efficiency of neoadjuvant chemotherapy administered to CC patients with metastatic LNs [5]. Accurate preoperative detection of metastatic LNs, therefore, is critical for generating an effective and individualized treatment plan for CC patients.

Computed tomography (CT) is an initial diagnostic tool for the evaluation of CC disease in clinical examination [5]. Despite well performance for assessment of T-stage of tumor using this imaging modality, diagnostic accuracy for regional metastasis is only 54% for the patients using current diagnostic criteria based on size of the LNs (metastatic LNs > 10 mm) [6] which demonstrates the unreliability of using the size to evaluate regional metastases in CC patients [7]. Although the clinicopathological characteristics demonstrate the potential for diagnosis of LN metastases [8–10], the requirement of surgical resection or biopsy for confirmation limits their usage in clinical practice. Therefore, novel approaches that use conventional CT imaging data to detect regional metastases are needed to develop better preoperative treatment planning for CC patients.

Radiomics is an emerging translational field of research that aims to describe tissue characteristics extracting high throughput quantitative features or biomarkers from multi-modality medical imaging data [11, 12]. Due to the ability to reveal complex intra-tumor heterogeneity, radiomics is considered to be a powerful tool in modern medicine including diagnosis, tumor characterization and prognosis [13]. In recent years, it has been used for the diagnosis of metastatic LNs in bladder, lung, biliary-tract, and esophagus cancers with satisfactory results [14–17]. However, a small number of these studies benefit from pathological verification and there is a paucity of quantitative analysis in CC to predict metastasis of regional LNs.

The purpose of this study was to develop and validate machine learning models that utilize either patient-characteristics or quantitative CT texture features for preoperative accurate diagnosis of metastasis in regional LNs and to compare their performance with clinical diagnosis criteria for LNs in CC patients.

## Methods

### Patients

**Fig. 1:**
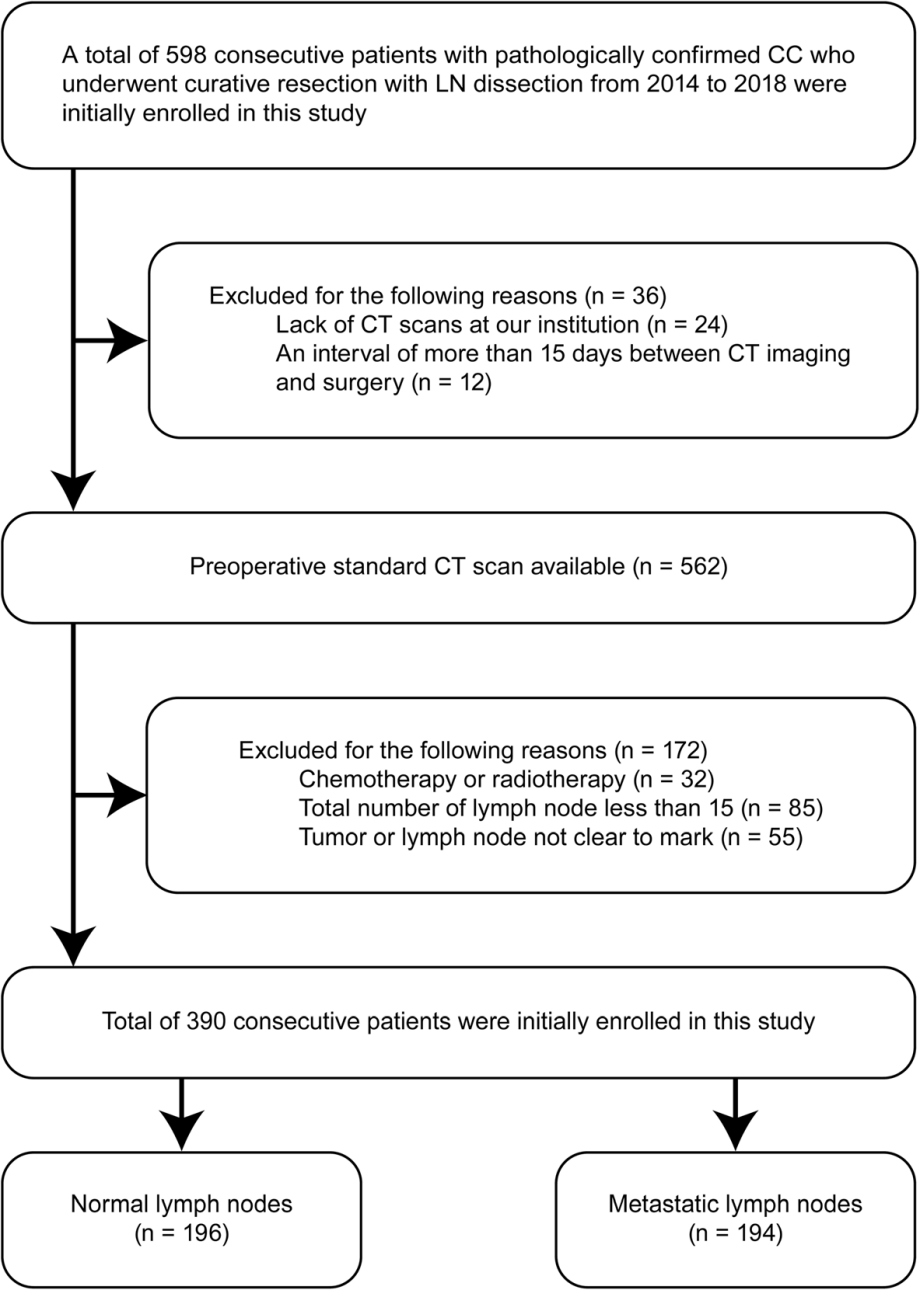
Recruitment pathway for patients in this study

A total of 390 patients were selected from the patient database of single-institution for this retrospective study among 598 patients who were diagnosed with CC and received colectomy with the removal of regional LNs from January 2014 to May 2018 in the Affiliated Hospital of Qingdao University. The detailed patient recruitment pathway with inclusion and exclusion criteria is described in Fig. 1.

Clinical data, including age, gender, and primary tumor site, were collected by reviewing medical records. Histological grade, T-stages, nerve invasion, and vessel invasion were obtained directly from pathological reports. The stage of the tumors was determined by surgical oncologists according to the American Joint Committee on Cancer (AJCC) TNM staging system, 8th edition [18].

### CT image acquisition

The CC patients were scanned before radical resection of colon tumors using Siemens Somatom Sensation 64 CT scanner (Siemens Medical Solutions, Erlangen, Germany). The CT imaging parameters were selected by radiologists and kept the same for all the patients which were 120 kV; 200 effective mAs; beam collimation of 64 mm × 0.6 mm; a matrix of 512 × 512; a pitch of 0.8; and a gantry rotation time of 0.5 s. The CT image data were reconstructed with a slice thickness of 5 mm and resampled using b-spline interpolation to set the in-plane resolution to 1 mm before the feature extraction process.

### Lymph node labeling and segmentation on CT data

After preoperative CT data acquisition, tumors and regional LNs were evaluated according to their anatomical regions as a standard procedure before the surgery. During the surgery, both tumor and LNs were removed and the dissected LNs were separated into different groups according to anatomical location. Following the surgery, all the resected LNs were sent to a pathology laboratory for histological analysis. The morphological and histological features of the LNs were recorded as metadata on patient records after pathological analysis. We generated a patient cohort using histology reports and preoperative CT data with LN markings to identify the location and the histological status of the LNs. In order to ensure the validity of the LN histology, the largest and most adjacent LNs to tumors were selected. Afterward, the largest LN from each patient was manually outlined to generate a region of interest (ROI) on the slice with maximal in-plane diameter using ITK-SNAP software [19] by an experienced radiologist. Later, these ROIs were validated by a senior radiologist in abdominal radiology.

### Feature extraction, selection, and model building

**Fig. 2:**
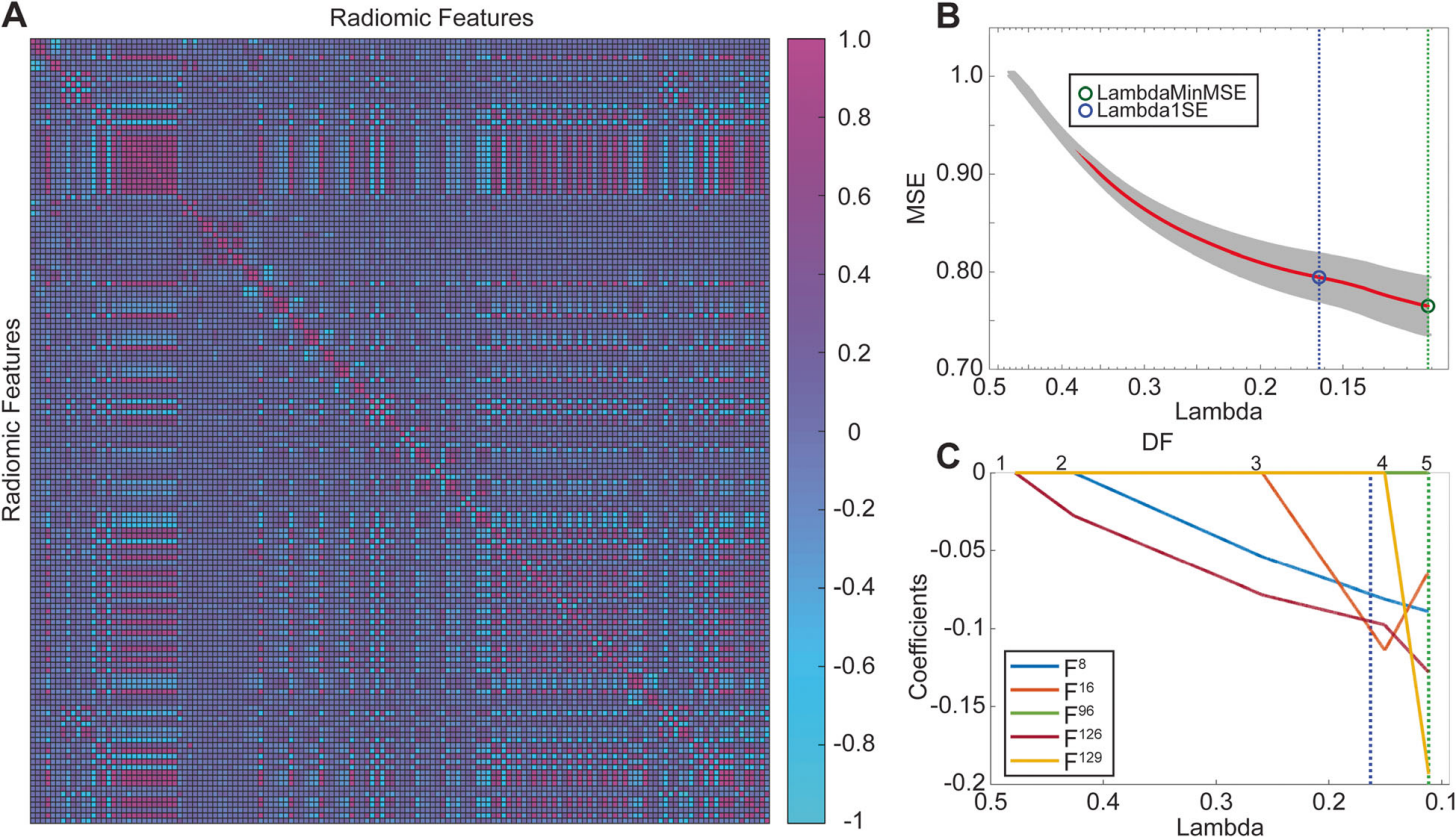
The correlation of the textural features and selection of a subset of features of lymph nodes using the least absolute shrinkage and selection operator regularization. Abbreviations: DF, Degree of freedom; F^8^, Contrast; F^16^, Run percentage; F^96^, Low gray level run emphasis of approximate wavelet image; F^126^, Contrast of gradient image; F^129^, Entropy of the gradient image

Before computing the features, CT data was quantized using a fixed number of bin sizes (8 bins) and rescaled into the range of [0,1] using min-max normalization. The quantitative CT image features were computed by employing six feature extraction methods, e.g. first-order statistics (FoS) (six features), gray-level co-occurrence matrix (GLCM) (six features), gray-level run-length matrix (GLRLM) (seven features), local binary pattern (LBP) (ten features), fractal analysis (FA) (one feature) and shape features (nine features). The FoS were computed to summarize the distribution of the intensity values of the CT image data regardless of spatial positioning [20]. Besides, GLCM features were computed for analysis of the tissue texture evaluating the spatial relationship of the voxels and GLRLM features were utilized to interpret coarseness of the texture by computing in four directions (0°, 45°, 90°, 135°) [21, 22]. Afterward, GLCM and GLRLM features computed for each direction were merged by averaging into a vector. Local binary patterns were used to describe local spatial patterns of intensity images while fractal analysis was performed to measure the rate of changing complexity of the texture with scale variation [23, 24]. The shape features were computed to interpret the structural characteristics of the tissues from generated ROIs. Besides, two image filters were utilized to capture the texture characteristics of the LNs in wavelet and gradient domains. The wavelet coefficient images were computed using Daubechies kernel function for analysis of the localized characteristics of the images at different scales while gradient images are the measurement of the directional changes of the image intensity [25]. FoS, GLRLM and GLCM features were computed from first level wavelet decomposition images (eighty-eight features). Besides, FoS, GLCM, and GLRLM features were also extracted from gradient images (nineteen features) to capture phenotypic details of tissues. A total of 146 features were extracted from preoperative CT data to reveal complex patterns of LN structures using in house developed scripts in Matlab® (v9.1.2, MathWorks, MA). The correlation of the features was demonstrated in a heat map representation in Fig. 2a. A regression model was generated to determine potential features using the least absolute shrinkage and selection operator (LASSO) algorithm with 10-fold cross-validation [26]. The variables, included in the regression model (variables with non-zero weights), were used to generate a classifier for the diagnosis of metastatic LNs. The behavior of the cross-validation mean-squared error was shown in Fig. 2b. Moreover, Fig. 2c demonstrated the variation of the weights of the features while minimizing the cross-validation error.

After extraction of the quantitative CT image features, the patients were randomly separated into two groups by keeping the same distribution of the metastatic and normal LNs in training (80%) and test cohorts (%20). The training cohort consisted of 312 patients with 155 metastatic and 157 normal LNs while 78 patients with 39 metastatic and 39 normal LNs included in the test cohort. The patients in the training cohort were used to optimize the classification models within a 10-fold cross-validation framework and the test cohort was only used to evaluate the performance of the final classification models.

The clinical model was generated employing LN size computed from ROIs drawn by the experienced radiologist. The LNs with a size of more than 10 mm was assumed as metastatic and remaining LNs as normal by following the clinical diagnosis approach.

The patient-demographic model was generated after analysis of patient characteristics acquired preoperatively (age, gender, histological grade, location, short- and long-axis diameters of LNs). The key features were selected according to the performance of the 10-fold cross-validation error of the generated classifiers during training and selected key features were used to generate the patient-demographic model.

The radiomic-derived model was constructed using the selected radiomics features (contrast and run percentage of intensity image, low gray level run emphasis of approximate wavelet image, contrast, and entropy of gradient image) with the LASSO algorithm of the patients in the training cohort by performing 10-fold cross-validation for performance evaluation of the generated model [27]. The patients in the test cohort were only used to evaluate the performance of classification for the diagnosis of metastatic LNs.

The diagnostic efficiency of these models was assessed using pathological reports of LNs in terms of accuracy, specificity, sensitivity, and area under the receiver operating curve (AUC) metrics. The AUC values were presented with 95% confidence interval and statistical difference among the generated models was evaluated using the DeLong method [28].

### Statistical analysis

The categorical demographic characteristics of the patients were evaluated with the binomial test using GraphPad Prism (v7.0, La Jolla, CA). For numerical clinical variables, the Wilcoxon rank-sum test was utilized to investigate the statistical significance between patients with normal and metastatic LNs. *p* < 0.05 was accepted as statistically significant. The variables were presented as mean ± standard deviation.

## Results

### Patient characteristics

A total of 390 patients (390 LNs) were incorporated in this study which 312 patients (157 normal and 155 metastatic LNs) were used in training cohort and 78 patients in test cohort (39 normal and 39 metastatic LNs). The area of the normal LNs was measured as 90.49 ± 114.04 mm^2^ (Median: 55, Range: [10, 816]) while metastatic LNs had an area of 185.31 ± 224.51 mm^2^ (Median: 122, Range: [12, 1724]). The clinicopathologic characteristics of patients are summarized in Table 1. There were no statistically significant differences in gender, age, location and histological grade of the tumor, however, perineural invasion, vascular invasion, and T-stage demonstrated a statistically significant difference (*p* < 0.05) between the patients with normal and metastatic LNs.

**Table 1:** Characteristics of patients with normal and metastatic LNs

Characteristics	Training Cohort			Validation Cohort		
	Patients with normal LNs (*n* = 157)	Patients with metastatic LNs (*n* = 155)	*p*	Patients with normal LNs (*n* = 39)	Patients with metastatic LNs (*n* = 39)	*p*
Age	63.89 ± 12.38	61.76 ± 12.45	0.133	62.56 ± 14.17	62.13 ± 13.25	0.890
Gender			0.076			0.120
Male	52.87%	56.13%		51.28%	38.46%	
Female	47.13	43.87%		48.72%	61.54%	
Tumor location			0.060			
Left	45.22%	50.32%		46.15%	46.15%	
Right	54.78%	49.68%		53.85%	53.85%	
Histological status			0.069			0.148
Well	67.52%	63.23%		71.80%	64.10%	
Poor	32.48%	36.77%		19.10%	35.90%	
Perineural invasion			< 0.01			< 0.01
Negative	73.89%	49.03%		76.92%	43.59%	
Positive	26.11%	50.07%		23.08%	56.41%	
Vessel invasion			< 0.01			< 0.01
Negative	94.27%	42.58%		92.31%	48.72%	
Positive	5.73%	57.52%		7.69%	51.28%	
T stage			< 0.01			< 0.01
T_1_	1.27%	0.65%		0%	0%	
T_2_	11.47%	1.94%		10.26%	0%	
T_3_	76.43%	70.31%		79.48%	61.54%	
T_4_	10.83%	27.10%		10.26%	38.46%	

### Performance evaluation

**Fig. 3:**
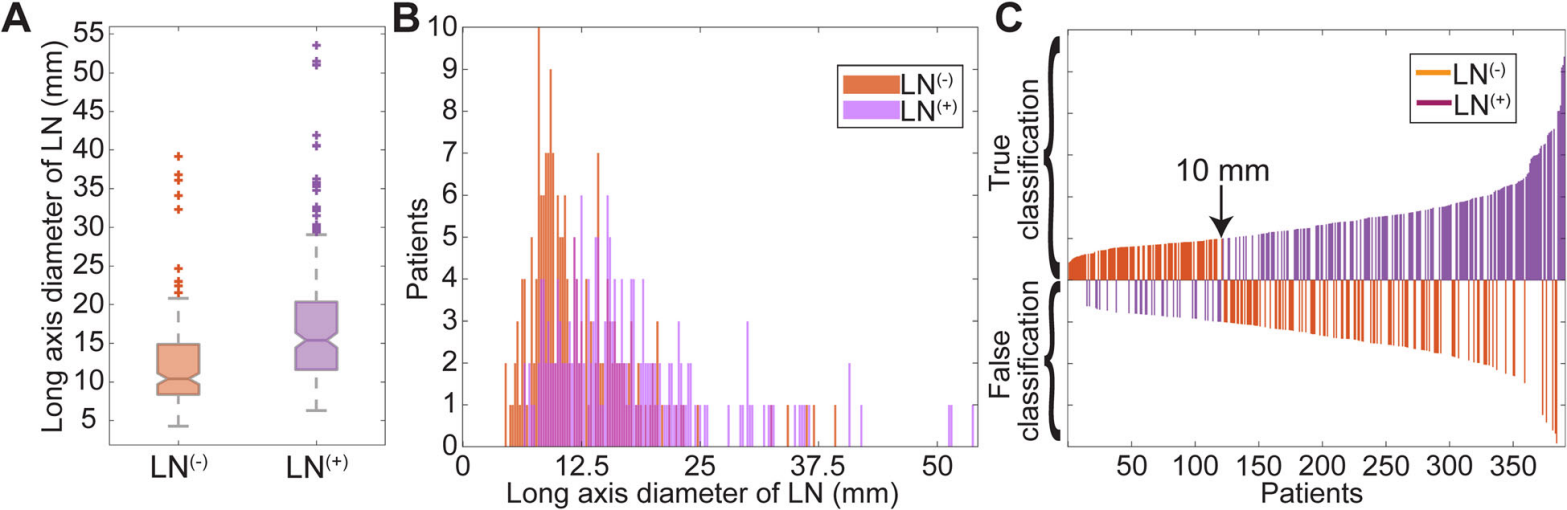
Evaluation of lymph nodes using current CT image diagnostic criteria

**Fig. 4:**
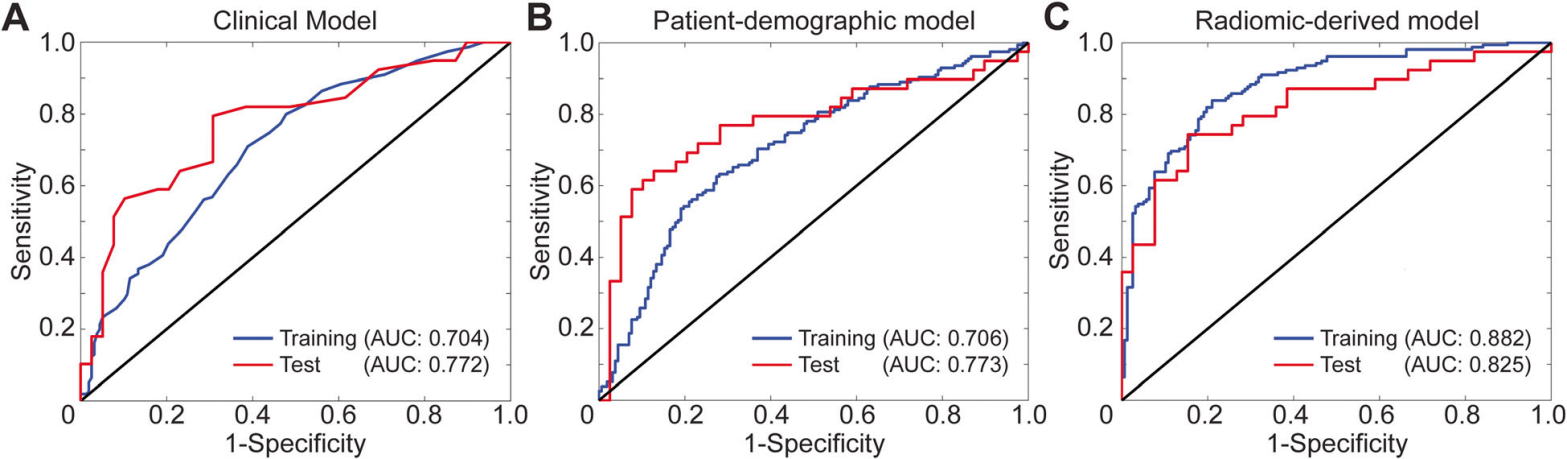
Receiver operating characteristics curves of the CT image diagnostic criteria, clinical and radiomics models for training and test cohorts

In the clinical model, metastatic LNs were differentiated from normal LNs by evaluating the diameter of the LNs in the direction of the longest axis. In our patient cohort, including patients selected for training and test process, had a long-axis diameter of 12.12 ± 5.74 mm for normal LNs while metastatic LNs had a diameter of 17.37 ± 8.48 mm (Fig. 3a). Wilcoxon rank-sum test showed that the long-axis diameter of the metastatic LNs was statistically different from normal LNs with a *p* < 0.01 (95% confidence interval [CI]: 3.81, 6.70]. However, the histogram of LNs with a resolution of 0.25 mm demonstrated that 74.87% of normal and metastatic LNs were clustered together in the same bins (Fig. 3b); therefore, 64.87% of the LNs were diagnosed correctly using clinical diagnostic criteria that correspond to correct classification of 253 LNs (204 and 49 LNs in training and test cohorts, respectively) in 390 CC patients (Fig. 3c). Specifically, 65.38% of the patients in the training cohort had a correct diagnosis while the diagnostic performance for the test cohort was 62.82%. Besides, the model had an AUC of 0.704 (95% CI: 0.675, 0.733) for training and 0.772 (95% CI: 0.718, 0.825) for test cohorts (Fig. 4a). The clinical model obtained a sensitivity of 83.87% and 84.62% for training and test cohorts while the specificity of 47.13% and 41.03% was observed for training and test cohorts, respectively (Table 2).

**Table 2:** Predictive performance of the CT diagnostic criteria and generated classifiers

Models	Dataset	Accuracy (%)	Sensitivity (%)	Specificity (%)	AUC ± 95% CI
Clinical model	Training	65.38	83.87	47.13	0.703 ± 0.03
	Test	62.82	84.62	41.03	0.772 ± 0.05
Patient-demographic model	Training	67.31	62.58	71.97	0.706 ± 0.03
	Test	73.08	69.23	76.92	0.773 ± 0.05
Radiomic-derived model	Training	81.09	83.87	78.34	0.882 ± 0.02
	Test	79.49	74.36	84.62	0.825 ± 0.05

After the evaluation of six features of CC patients, the short-axis diameter of LN had the best classification accuracy with the least cross-validation error. Therefore, the patient-demographic classification model was generated using the short-axis diameter of LNs. The model demonstrated an accuracy of 67.31% for training and 73.08% for the test data which corresponds to the correct classification of 267 LNs (210 and 57 LNs in training and test cohorts, respectively). Moreover, the model had a sensitivity of 62.58% for training and 69.23% for the test data while obtaining a specificity of 71.97% and 76.92% for training and test set, respectively. This model showed an AUC of 0.706 (95% CI: 0.677, 0.735) for training and 0.773 (95% CI: 0.720, 0.827) for test data (Table 2). There was no statistically significant improvement compared to the clinical model for training and test cohorts (*p* = [0.982, 0.997]). The classifier performance for training and test cohorts are presented in Fig. 4b.

The key features for the radiomic-derived model were determined by performing LASSO regularization with 10-fold cross-validation among 146 CT image features. The selected five features were used to build a radiomic-derived classification model. The radiomic-derived model demonstrated better performance for training (81.09%) and test cohorts (79.49%) in terms of accuracy with an increase of over 15% compared to the CT-image diagnostic criteria that corresponded to an additional 63 accurately diagnosed LNs. Besides, the model correctly identified a total of 315 LNs combined of 253 LNs from the training cohort and 62 LNs from the test cohort. The sensitivity of the training cohort was higher than the clinical model but similar to the clinical model while the radiomic-derived model generated lower sensitivity for the test cohort compared to CT-image diagnostic criteria but higher than the patient-demographic model. Specificity was 78.34% for training and 84.62% for the test cohorts. In addition, the classifier model showed a significant increase in AUC for training (17.6%, *p* < 0.001) and test groups (5.2%, *p* < 0.02) resulting in an AUC of 0.882 [95% CI: 0.862, 0.901] for training and 0.825 [95% CI: 0.778, 0.872] for the test cohorts. Figure 4c portrays the performance of the model for the training and test sets. Moreover, Table 2 summarizes the prediction performance of the three models**.**

## Discussion

In our study, we compared the diagnostic accuracy of clinical criteria to detect metastatic LNs in CC patients with two classification models such that the patient-demographic model utilizing short-axis LN diameter and radiomic-derived model incorporating five texture features of preoperative CT data. Our results demonstrated that the radiomic-derived model had significantly better performance compared to the clinical diagnostic criteria and patient-demographic model for the detection of normal and metastatic LNs in CC patients (Table 2).

Metastatic LN plays a crucial role in preoperative stage evaluation and development of treatment planning. In clinical practice, metastatic LNs are identified based on long-axis diameter size during the evaluation of CT images [29–31]. However, the diagnostic performance of LNs in clinical studies is widely affected due to unreliability of LN size for diagnosis of nodal metastasis in CC [32] such that a clinical study demonstrated that metastatic LN was detected with an accuracy of 54% in CC patients using CT [6]. In our study, we used clinically accepted diagnostic criteria for metastatic LNs (> 10 mm) to evaluate the detection performance of metastatic LNs [7]. The histogram with a resolution (width size of the bin) of 0.25 mm demonstrated that 298 LNs (normal or metastatic) were clustered in the same bins resulting 74.87% overlap; therefore, clinical diagnosis criteria had a correct classification for 253 LNs among 390. Other studies investigated morphological characteristics of LNs e.g. visible internal heterogeneity and irregular boundary, to differentiate normal and metastatic LNs in CC patients that improved the specificity from 63% to 73%, however, the long-term effect is still being investigated [33–35]. Due to the limited presence of visible heterogeneity and irregularity of boundary for metastases on CT images, Rollven et al. suggested that morphological CT criteria are not sufficient for nodal staging [36]. Therefore, better tools are urgently needed to accurately diagnose LN metastasis preoperatively.

Radiomics, computing high-dimensional quantitative features, has demonstrated potential benefits for different types of applications e.g. diagnosis, prognosis, prediction of treatment outcomes or overall survival. Ji et al. developed a radiomic signature interpreting the quantitative features of preoperative CT data to diagnose metastasis LN in biliary tract cancer which was determined with a blood test [14]. The model had an AUC of 0.81 for training and 0.80 for validation cohorts. Besides, Shen et al. developed a multivariable model to diagnose LN status for esophageal cancer patients by utilizing CT-reported LN metastasis status, CT-reported positions and 13 texture-based features. The model obtained an AUC of 0.806 and 0.771 for training and test cohorts, respectively. Despite other studies focusing on the prediction of metastatic LNs in several cancer types [14–17], there is still a paucity of research that integrates radiomics features with machine learning to diagnose metastatic LNs with pathological validation [37]. In this study, we developed two machine learning models, e.g. patient-demographic utilizing selected features from patient characteristics and radiomic-derived model benefiting of quantitative imaging features computed from preoperative CT data, and compared with the clinical diagnosis criteria (clinical model) for LN metastasis. While the clinical model (AUC of 0.772) and the patient-demographic model (AUC of 0.773) had similar diagnostic accuracy for the test cohort (*p* = 0.987), the radiomic-derived model obtained a statistically significant improvement in diagnostic performance (AUC of 0.825, *p* < 0.02). Specifically, 226 LNs were correctly classified by radiomic-derived and clinical model, while 89 LNs detected with the radiomic-derived model only and 27 LNs by clinical model. Besides, there were 48 LNs were misclassified by both radiomic-derived and clinical models).

There were several limitations to our study. It was a retrospective study that included only the largest regional LN from each patient to obtain pathological validation; therefore, our findings will benefit from a prospective study designed to collect multiple LNs from each patient. Due to performing a monocentric study, we could not evaluate the reproducibility of the radiomics features that may be affected by the acquisition parameters of the monocentric study design. Therefore, multicenter studies with different CT data acquisition parameters may improve the performance of the diagnosis with the assessment of the reproducibility of these features. Additionally, the ROIs of the LNs were drawn and validated using a manual approach by two experienced radiologists. Although manual segmentation is a commonly implemented approach in clinical studies, the implementation of automated segmentation would decrease the time required for the preparation of data. Finally, our study lacks postoperative follow-up data, so we could not examine the relationship between the texture of CT data and survival outcomes. Future studies are needed to evaluate the correlation between LN image biomarkers and overall survival.

## Conclusion

Our study demonstrated that a radiomics model can be used to detect metastatic LNs preoperatively in CC patients, which can improve the diagnostic accuracy compared to the current clinical standard for diagnosis of nodal metastasis. The kernel-based SVM classification model had significantly better diagnostic performance than clinical and patient-demographic models. The findings of our study may be helpful for the selection of suitable treatment approaches for CC patients to improve the survival rates of the patients.

## Data Availability

The datasets generated during and/or analyzed during the current study are available from the corresponding author on reasonable request.

## Abbreviations

AJCC: American joint committee of cancer
AUC: Area under the receiver operating curve
CC: Colon cancer
CI: Confidence interval
CT: Computed tomography
FA: Fractal analysis
FoS: First order statistics
GLCM: Gray level co-occurrence matrix
GLRLM: Gray level run-length matrix
LBP: Local binary patterns
LN: Lymph node
LASSO: Least absolute shrinkage and selection operator
RBF: Radial basis function
ROI: Region of interest
SVM: Support vector machines
